# Online Pedagogical Tutorial Tactics Optimization Using Genetic-Based Reinforcement Learning

**DOI:** 10.1155/2015/352895

**Published:** 2015-05-07

**Authors:** Hsuan-Ta Lin, Po-Ming Lee, Tzu-Chien Hsiao

**Affiliations:** ^1^Institute of Biomedical Engineering, National Chiao Tung University, 1001 University Road, Hsinchu, Taiwan; ^2^Institute of Computer Science and Engineering, National Chiao Tung University, 1001 University Road, Hsinchu, Taiwan; ^3^Department of Computer Science, National Chiao Tung University, 1001 University Road, Hsinchu, Taiwan; ^4^Biomedical Electronics Translational Research Center and Biomimetic Systems Research Center, National Chiao Tung University, 1001 University Road, Hsinchu, Taiwan

## Abstract

Tutorial tactics are policies for an Intelligent Tutoring System (ITS) to decide the next action when there are multiple actions available. Recent research has demonstrated that when the learning contents were controlled so as to be the same, different tutorial tactics would make difference in students' learning gains. However, the Reinforcement Learning (RL) techniques that were used in previous studies to induce tutorial tactics are insufficient when encountering large problems and hence were used in offline manners. Therefore, we introduced a Genetic-Based Reinforcement Learning (GBML) approach to induce tutorial tactics in an online-learning manner without basing on any preexisting dataset. The introduced method can learn a set of rules from the environment in a manner similar to RL. It includes a genetic-based optimizer for rule discovery task by generating new rules from the old ones. This increases the scalability of a RL learner for larger problems. The results support our hypothesis about the capability of the GBML method to induce tutorial tactics. This suggests that the GBML method should be favorable in developing real-world ITS applications in the domain of tutorial tactics induction.

## 1. Introduction

*Intelligent Tutoring Systems*. Human one-to-one tutoring is one of the most effective educational interventions in that tutored students often perform significantly better than students in group instructional methods [1]. The development of computer has enabled teachers to overcome the problem of educating students who originally are unable to take advantage of one-to-one tutoring due to cost and time constraints. Computer-based training (CBT) and computer aided instruction (CAI) were the first such systems developed as an attempt to tutor. While both CBT and CAI may be somewhat effective in helping learning, they may not be adequate to provide the same level of individualized attention that a student would receive from a human tutor. In order to make tutorial decisions effective, it is assumed that tutors should adapt their actions to students' needs based upon students' current knowledge level and emotional states [2]. Researchers adopted the human tutor as their educational model and sought to apply artificial intelligence techniques to realize this model in “intelligent” computer-based instruction. This has prompted research in the field of Intelligent Tutoring System (ITS) [3–5].

**Figure 1:**
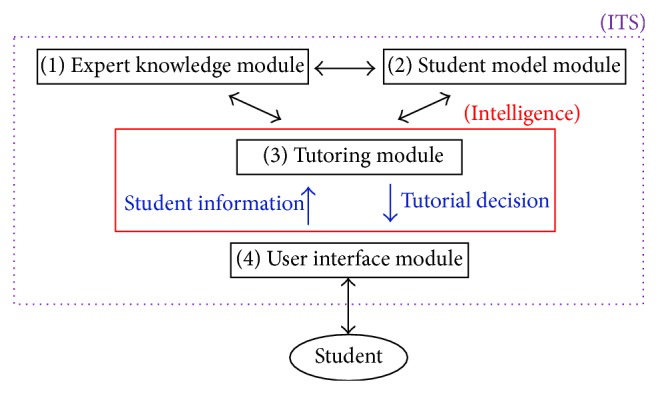
A general architecture of an ITS.

An ITS is a computer system that aims to provide immediate and customized instruction or feedback to learners [6]. An ITS consists of four basic components based on a general consensus amongst researchers.  Figure 1 illustrates the general form of an ITS architecture. The expert knowledge module contains the concepts, rules, and problem-solving strategies of the domain to be learned. The student model module stores information that is specific to each individual learner. It should contain as much knowledge as possible about the student's cognitive and affective states. The purpose of the student model module is to provide data for the tutoring module of the system. The tutoring module provides a model of the teaching process and makes decisions about tutoring strategies and actions. The dialogue and screen layouts are controlled by the user interface module. An ITS may then achieve the “intelligence” by presenting tutorial decisions based on the expert knowledge module and the information about the learner. On the research path of the ITS, numerous applications have been developed and applied to real world, especially in high schools and universities. For instance, the cognitive tutor has been incorporated into mathematics curricula in a substantial number of United States high schools, producing improved student learning outcomes on final exams and standardized tests [7, 8].

*Tutorial Tactics Optimization*. Tutorial tactics optimization is an essential issue of developing an ITS [9], since the tutorial tactics can largely affect the learning performance even when the learning contents are the same. To optimize the tutorial tactics, Reinforcement Learning (RL) is often used in the field instead of hard coding. The RL is an approach of machine learning (ML) technique that aims at learning what to do in a previously unknown scenario by maximizing the obtained reward provided by its user. In the RL, an agent must be able to sense the state of the environment and to take actions that affect the state [10]. The agent interacts with the environment on a sequence of discrete time step. At each time step, the agent receives condition information from the environment, selects an action, and then receives a reward of the action. However, a typical RL algorithm, for instance, Q-learning (QL) [11], may have the following issues. The first issue is feature selection [9, 12]. Increasing the size of the state space makes the collected data much sparser sampling of the domain. The second issue is the explore-exploit dilemma [13]. The agent has to exploit what it already knows in order to obtain reward, and it also required to explore more in order to make better action selections in the future.

Although the RL methods have been applied to numerous conventional ITSs, no attempt in “online-learning” has been conducted due to the restriction of the RL method. Online learning here stands for online ML, which is used in the case where the data becomes available in a sequential fashion, to determine an optimal action that leads to a desired result. The key difference between online learning and batch learning (or “offline” learning) techniques is that in online learning the mapping is updated after the arrival of every new data point in a scalable fashion and can determine the optimal action at any time during the model building process, whereas batch techniques have to build a model from the entire training dataset before starting to have the model determining the optimal action. Due to the reason mentioned, the current study attempted to achieve the “online learning” by adding Genetic-Based Reinforcement Learning (GBML) algorithm to a step-based TS [5] called Tempranillo [14]. Specifically, the extended classifier system (XCS) was used in our study to induce tutorial tactics in an online-learning manner without having any preexisting datasets. The induced tutorial tactics of the XCS were evaluated on real human subjects.

*Objective and Contributions*. The objective of the current study is, by taking Tempranillo as an instance, to demonstrate that “online” tutorial tactics optimization can be achieved. The contribution of the current study is that, for the first time, the “online” tutorial tactics optimization is proved to be achievable and the procedure is reported. Of our best knowledge, no studies have achieved this before. In previous studies, researchers have to (1) collect data for a long time (i.e., months to years) to obtain sufficient data for model building, (2) build a model by using the ML algorithms, and then (3) apply the built model for the future use (e.g., next experiment). But the current study successfully built an ITS that dynamically optimized its tutorial tactics at the same time it was being used by students. This greatly improved the scalability of an ITS, since sample size of data and data collection are no longer problems for model building. Researchers can build such ITS and deploy it as soon as it is built. The ITS with the capability of online learning can improve its performance as time passes.

## 2. Literature Review

Beck et al. [15] applied the RL to make tutorial decision that caused students to spend less problem-solving time. However, the authors used simulated data but not data collected in real world as the training dataset. Chauncey and Azevedo [16] used a false-biofeedback paradigm to induce emotional states in college students while they used a multimedia learning environment. In the false-biofeedback paradigm, the goal is to induce emotions by causing individuals to believe that they are experiencing physiological arousal. The study indicated that the induced emotional states can affect students' metacognitive judgments. Chi [9] applied the RL to induce two sets of tutorial policies from preexisting human interaction data. The normalized gain (NormGain) set was derived with the goal of enhancing tutorial decisions that contribute to learning. The reverse normalized gain (RevNormGain or “InvNormGain”) set was derived with the goal of enhancing tutorial decisions that contribute less or even nothing to learning. This study indicates that when the learning contents were controlled so as to be the same, different tutorial tactics would make difference in students' learning gains (based on their immediate posttest scores). Olney et al. [17] found significant difference in learning gains of students who used the ITS compared to the users under group instruction. Baker et al. [18] aimed to improve the tutorial tactics optimization by building novel student models to determine exactly at which point a knowledge component (KC) [19] is learned (i.e., identifying the “moments of learning”). Other ML approaches other than RL have also been tested in the path of research of tutorial tactics optimization [12]. For instance, Ezen-Can and Boyer [20] tested the unsupervised ML methods for classification of student dialogue. Mitchell et al. [21] presented a Markov decision process (MDP) framework to learn an intervention policy capturing the most effective tutor behaviors in a task-oriented learning environment with textual dialogue. The built system was found effective in maintaining student engagement. Whitehill [22] built ITS that optimizes tutorial tactics based on effect related data and a stochastic control framework similar to the one used in [21]. The study reported that they improved the students' learning process in engagement and their perception of curriculum difficulty.

Becker et al. [24] extended the study [9] that tested the effectiveness of RL in tutorial tactics optimization in ITS, by applying expert-guided learning approach to the model building process. The authors ranked the candidate questions as the follow-up for dialogue-based ITS in tutoring based on the judgments collected from expert human tutor. Rowe and Lester [25] extended the study [9] by proposing a modular RL framework in an implementation of interactive narrative system. The proposed framework involves decomposing an interactive narrative into multiple independent MDPs. Policies for each MDP were induced using a dataset of user interaction data collected from an interactive narrative system. The results obtained show that the proposed framework was effective. Later, Ramachandran and Scassellati [26] proposed to add the RL to a social robot ITS for adaptively customizing the pace and content of a student's curriculum. However, the results were not yet reported. The study [27] that aimed to identify the effective moves in tutoring for tutorial dialogue systems also uses fine-grained level analysis (finer grain level analysis means increasing the resolution in the analysis; the tutorial tactics that the current study aimed to optimize are the microlevel (fined-grained) tutorial tactics, which means these tactics were optimized in a short-term time scale) as the current study did to optimize its tutorial tactics based in response to student actions. Lipschultz et al. [28] aimed to develop a fully automatic ML-learned computer-based tutor by using only automatically extractable features (e.g., percent of domain words in student turn) and features that are available in a tutoring system (e.g., correctness). However, the study focused on features searching but not the used ML algorithms as we did. To summarize, the current study is in line with the previous studies that represent a step toward the larger goal of tutorial tactics optimization directly and automatically from human examples. While it is crucial to achieve “online learning” in order to enhance the scalability of ITSs in tutorial tactics optimization, none of the studies has achieved “online learning.” This is the reason why the current study aimed to address this issue.

## 3. Materials and Method

### 3.1. Genetic-Based Reinforcement Learning Algorithm (the XCS)

**Figure 2:**
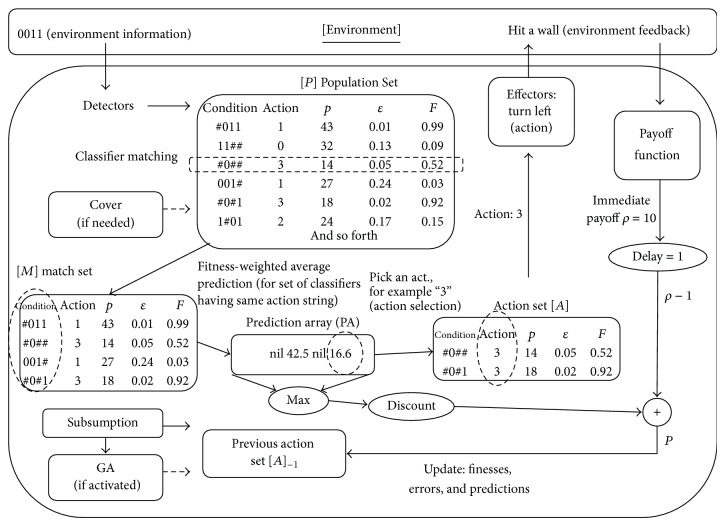
The system architecture of the XCS.

**Figure 3:**
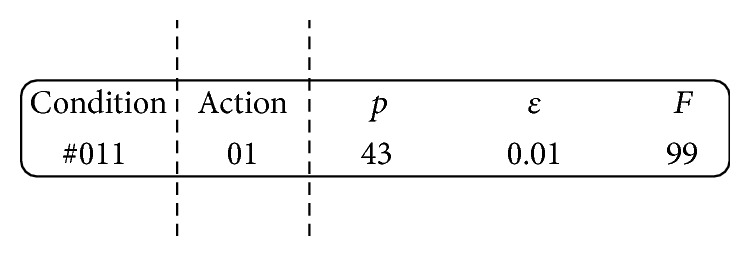
An example of a typical classifier managed by XCS.

The XCS is a rule-based online learning algorithm which is able to manage a set of classifiers that are represented in the traditional production system form of “IF state THEN action.” By integrating the genetic algorithm (GA) component (also named rule discovery component in XCS), the set of classifiers evolves occasionally and searches for a set of classifiers that yields the maximal generality and accuracy [29]. A detailed description on the flow of a typical XCS learning iteration is presented as follows. First, as shown in Figure 2, the XCS detector encodes the status of the environment into a binary string. Second, the XCS uses the binary string for matching classifiers in its rule set (denoted by [*P*]). As shown in Figure 3, a classifier managed by the XCS can be divided into three parts: a ternary string representing condition (for certain situations, 0, 1, and # for each bit, # indicates a bit that should be ignored, also called “don't care” bit), a binary string representing an action (the output suggested by the classifier) and three parameters of the classifier, in which *p* represents predicted payoff, *ε* represents prediction error, and *F* represents fitness value. While the original XCS was designed to perform on datasets with discrete inputs and a discrete output, numerous versions of the XCS were developed over the past decade to suit various types of problems [30]. Our study adopted the input representation similar to the one proposed in [30] to accept integers in the input string.

During the matching process, the XCS searches for classifiers in [*P*], in which the condition space represented by its condition string (0, 1, and # for each bit) includes the detected current status, and places all matched classifiers into the Match Set (denoted by [*M*]). If the XCS does not find any classifiers during classifier matching process, the XCS then applies Cover process to generate a new classifier to enable their condition string to match current input (i.e., the environmental status). In Cover process, the action of the classifier is chosen at random. Third, the XCS calculates the fitness weighted average prediction from sets of classifiers that suggest the same action after [*M*] is generated. After calculating *P*_*i*_ for each possible output, the prediction array (PA) is formed for output selection process. The output selection regime is usually set to pick up output *i*, which owns the maximal predicted payoff, max⁡⁡(*P*_*i*_), in the PA, and occasionally picks up an output for exploration purpose arbitrarily. Fourth, the Effectors then perform the selected action to the environment. After that is the Q-learning style RL part of the XCS (on the bottom right of Figure 2). After Effectors performed the action to the environment, feedback generated by the environment is gathered by XCS and translated to pay off through the payoff function. Payoff function is a function predefined by the user of the XCS to interpret the feedback into numeric form of payoff. The occurrence of parameters update happened in the next iteration, on the Action Set generated in the last iteration (i.e., previous Action Set [*A*]_−1_), after the calculation of reward *P* through the following formula: *P* = *ρ*_−1_ + *γ* × max⁡⁡(PA). The computed reward *P* is used to update parameters (*p*, *ε*, and *F*) of each classifier. The Update process is held on only the last Action Set [*A*]_−1_, which represents the set of classifiers (demonstrated in the right of Figure 2) that are responsible for the environmental feedback caused by the classifiers' suggested action performed by the Effectors. Finally is the rule (classifier) discovery (i.e., the GA) part of XCS (on the left bottom of Figure 2). The GA is triggered occasionally to search for accurate classifiers in the classifier (condition-action string representation) space.

During the Update process and GA, subsumption is performed to enable “macroclassifiers” (classifiers that are more general than others) to subsume other classifiers to reduce the number of redundant, overlapped classifiers. Subsumption and the macroclassifiers technique are used together in the XCS to reduce redundant classifiers and to improve the generalization capability of the XCS. By reducing redundant classifiers, the use of the macroclassifiers technique realizes the ability of XCS in generalizing classifiers and also increases the speed of classifier matching process. The concept of the macroclassifiers is typically implemented by using an additional parameter “numerosity” (usually denoted by num) [31], in which a classifier with numerosity num = *n* is equivalent to *n* regular classifiers. When the XCS generates a new classifier at the initialization step or at later stages, the [*P*] is scanned to examine whether a macroclassifier exists with the condition string representing the super set of the input space represented by the new classifier, and the action between the new classifier and the macroclassifier is equal. If that is the case, the numerosity of that macroclassifier is incremented by one instead of inserting the new classifier into [*P*]. Otherwise, the new classifier is added to the population with its own numerosity field set to one. Similarly, when the macroclassifier experiences a deletion, its numerosity is decremented by one instead of being deleted and then any macroclassifier with numerosity num = 0 is removed from population. This process is called “subsumption”; it occurs after the Update process of [*A*] and GA (also called Action Set Subsumption and GA Subsumption, resp.) [31]. During Action Set Subsumption deletion, the XCS selects an experienced classifier *G* with *ε* < *ε*_0_ first; subsequently, *G* subsumes all classifiers in [*A*] that are less general than *G* by deleting them. Then the numerosity of *G* is incremented accordingly. The XCS also operates GA Subsumption deletion when new classifiers (children) are generated through GA; the children are compared to their parent classifiers and subsumed as well if the parent classifiers are experienced and are more general. Simultaneously, the numerosity of the subsuming classifier is incremented. Otherwise, XCS inserts the generated new classifiers into [*P*]. For further details of XCS, it is recommended to refer to Butz's algorithmic description of XCS [31].

### 3.2. Architecture of the Proposed Intelligent Tutoring System

Within Tempranillo, students complete linear algebra (LA) problems and are formatively assessed based on a KC model [19], providing information about their knowledge to their teachers. In Tempranillo, a student needs to apply several domain principles (some of the principles may need to be applied multiple times) to solve a LA problem. Our work used the “linear transformations” and the “orthogonality” of LA domain as covered in the first-year college LA course. The ten primary KCs that were used in both the experiments were definition of equivalence (KC1), definition of strict triangular form (KC2), definition of row echelon form (KC3), definition of Gaussian elimination (KC4), definition of reduced row echelon form (KC5), matrix addition (KC6), scalar multiplication (KC7), matrix multiplication (KC8), identity matrix (KC9), and matrix inversion (KC10).

**Figure 4:**
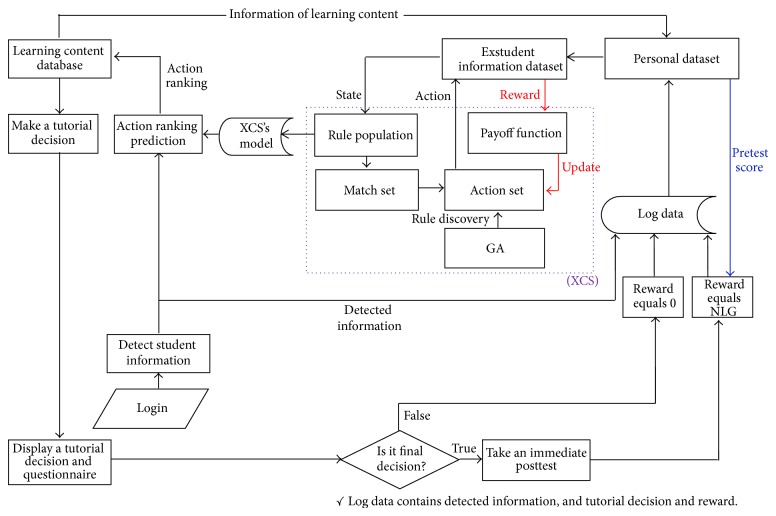
System structure of Tempranillo.

Figure 4 illustrates the system structure of Tempranillo. The XCS will online extract knowledge from an online-accumulated student interaction dataset and output the learned model. Tutoring module does an action ranking prediction by using XCS's model and makes a tutorial decision. Normalized learning gain (NLG) [9] was used as our payoff function since it measures students' gain irrespective of their incoming competence. The payoff function in XCS is implemented as follows:

(1) $$\begin{array}{c} \text{NLG}=\frac{\text{Posttest score}-\text{Pretest score}}{1-\text{Pretest score}}. \end{array}$$

This study excludes the situation of pretest score equal to 1 because of the restriction of the NLG formula. Pretest score and posttest score shown here refer to the students' test scores before and after the training, respectively. Notably, only the final decision in each learning topic has nonzero reward because a student's NLG will not be available until the posttest is completed. Our study identified thirteen attributes that are relevant for an ITS when making tutorial decisions. Three types of tutorial decisions (i.e., action of the agent): conceptual understanding, procedural knowledge, and problem-solving, are defined based on National Assessment of Educational Progress (NAEP) mathematics framework (National Assessment of Educational Progress (NAEP) by National Assessment Governing Board, U.S. Department of Education, USA; available at http://nces.ed.gov/nationsreportcard/).  Table 1 shows the relationship between state and module.

**Figure 5:**
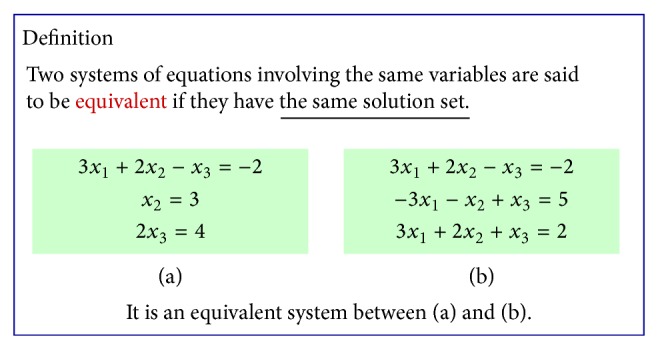
An example of conceptual understanding.

**Figure 6:**
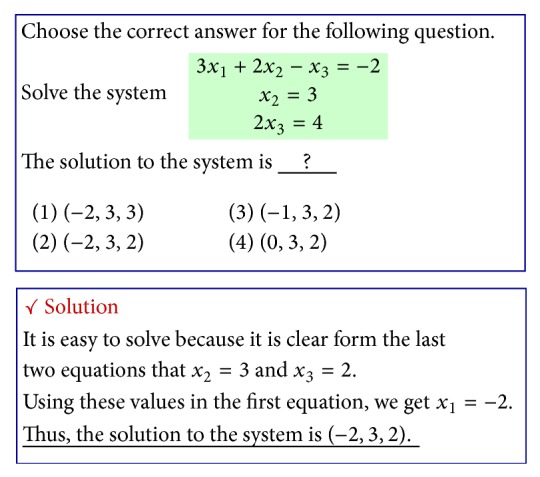
An example of procedural knowledge.

**Figure 7:**
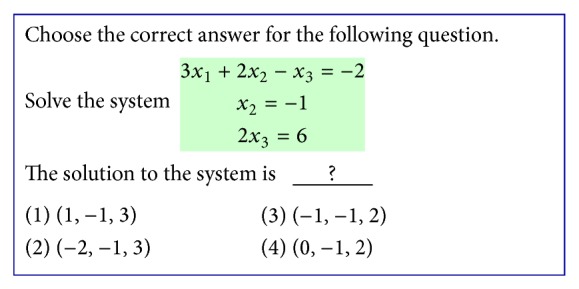
An example of problem-solving.

The following three types of tutorial decisions are defined based on NAEP mathematics framework. As shown in Figure 5, conceptual understanding is a comprehension of mathematical concepts. As shown in Figure 6, procedural knowledge is about rules and strategies to solve problems. As shown in Figure 7, the aim of teaching through problem-solving is to encourage students to refine and build onto their own processes.

### 3.3. Human Subject Experiment Done in Real World

We have conducted two human subject experiments in real world to prove that the Tempranillo is effective. Participants in experiment 1 were students who have learned LA for one semester, whereas participants in experiment 2 were students without prior knowledge of LA. In each experiment, the participants were randomly assigned to two groups, NormGain and RevNormGain as in [9] for evaluation of the performance of Tempranillo. The tutorial tactics of the ITS for the normalized gain (NormGain) group were optimized by using NLG as rewards for online model building whereas 1-NLG as rewards for the reverse normalized gain (RevNormGain) group. Other than the induced NormGain and RevNormGain tutorial tactics, the components of Tempranillo, including user interface, questionnaire, and tutorial scripts, were identical for all participants. Both the experiments were conducted in a double-blind manner.

### 3.4. Experimental Procedure

All participants (i.e., participants in NormGain group for experiment 1; RevNormGain group for experiment 1; NormGain group for experiment 2; and RevNormGain group for experiment 2) experienced an identical procedure. The procedure was as follows: (1) a background survey; (2) reading a textbook covering the target domain knowledge; (3) taking a pretest; (4) solving the same ten training problems in the same order on Tempranillo; and (5) taking a posttest. The pretest and posttest were identical. All tests were graded by a domain expert. A KC-based score for each KC application was given by identifying all relevant KCs over all test questions. In the following sections, the evaluation of the competence of each participant is provided based on the sum of all of these KC-based scores. The tests contained 36 test questions which cover 41 KC occurrences. All test scores were normalized to fall in the range of [0,1] for comparison purposes. The participants were not informed of the following before the experiment: how long the experiment would take, the experimental procedure, or whether the posttests would be administered.

## 4. Experiment  1: To Students with Prior Knowledge of LA

### 4.1. Participants

The dataset was collected over a period of four months during the fall and winter of 2014. There were 32 students ranging in age from 17 to 19 (*M* = 17.9; SD = 0.4; 24 men and 8 women) participating in experiment 1. The participants were college students selected from a university in Taiwan, who had just enrolled in an LA course for one semester, with normal or corrected-to-normal vision. They (1) were same in their age interval (SD = 0.4), (2) were same in domain knowledge they had, (3) were same in their college major, (4) were same in the length of time elapsing since they first learned LA, (5) were enrolled in the same class of LA before the experiment, and (6) were same in the LA text book used for the study in the class of LA. All the participants self-reported that they were nonsmoker, healthy, and with no history of brain injury and cardiovascular problems. Each participant took from two to three weeks to complete the study over multiple sessions.

### 4.2. Hypotheses

Specifically, the hypotheses that experiment 1 aimed to prove are as follows.

- H1.1: There was no significant difference (*p* < .05) between NormGain and RevNormGain group in their test scores before the use of Tempranillo, for students with prior knowledge of LA.
- H1.2: There was significant difference (*p* < .05) in the test scores of NormGain group before and after the use of Tempranillo, for students with prior knowledge of LA.
- H1.3: There was no significant difference (*p* < .05) in the test scores of RevNormGain group before and after the use of Tempranillo, for students with prior knowledge of LA.
- H1.4: There was significant difference (*p* < .05) between NormGain and RevNormGain group in their test scores after the use of Tempranillo, for students with prior knowledge of LA.

### 4.3. Results

**Figure 8:**
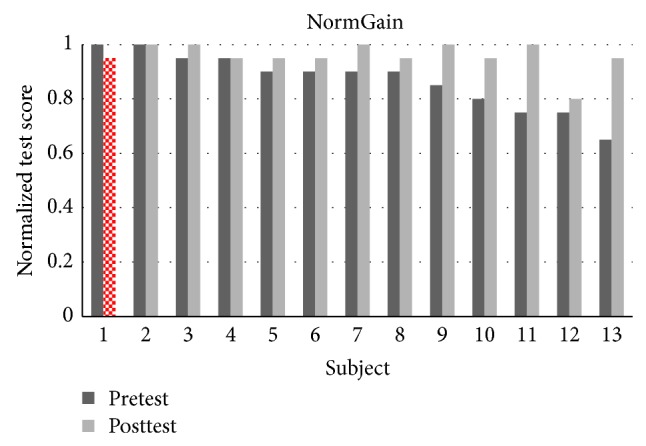
Normalized test score in experiment 1, NormGain group.

**Figure 9:**
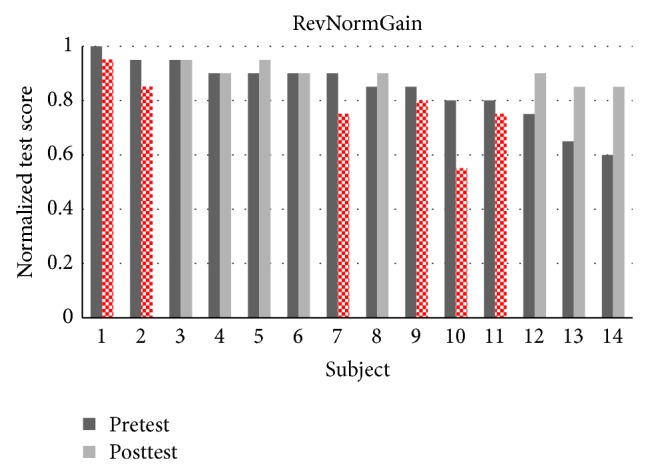
Normalized test score in experiment 1, RevNormGain group.

There were 32 students in experiment 1 where 27 students completed all the topics. There were 13 students in the NormGain subgroup and 14 students in the RevNormGain group.  Figure 8 illustrates normalized test score of the students in NormGain group and Figure 9 illustrates normalized test score of the students in RevNormGain group.

**Figure 10:**
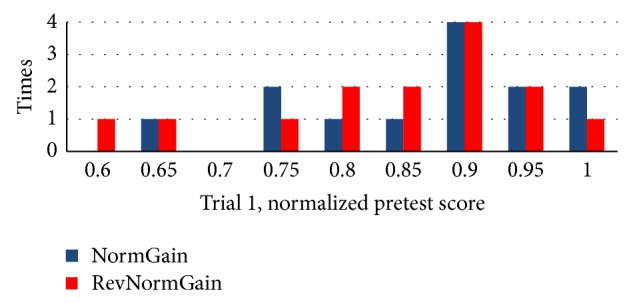
Normalized pretest score between two groups in experiment 1.

The histograms of students' pretest scores between two groups are shown in Figure 10.

**Figure 11:**
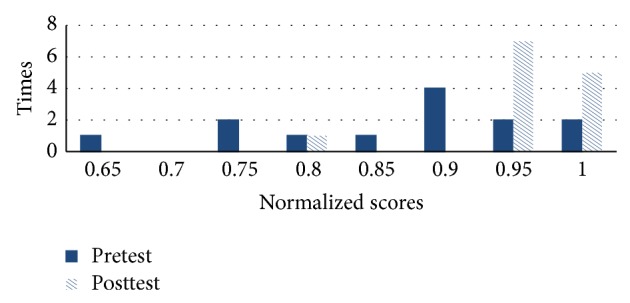
Normalized test score in experiment 1, NormGain group.

**Figure 12:**
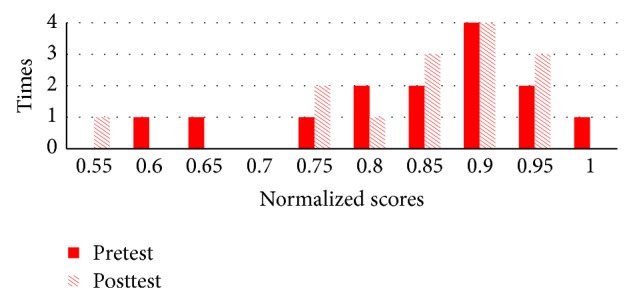
Normalized test score in experiment 1, RevNormGain group.

As shown in Figures 11 and 12, Figure 11 compares pretest and posttest scores of the students in NormGain group.  Figure 12 compares pretest and posttest scores of the students in RevNormGain group.

It is clear that the students' test scores are nonnormally distributed; the Wilcoxon rank sum test [32] was used for statistical tests in this study. Suppose that one has samples of observations from populations *A* and *B* containing *n*1 and *n*2 observations, respectively. The Wilcoxon test is based on the ranking of the observations of the combined sample (*n*1 + *n*2) in which each observation has a rank. For instance, the smallest observation has rank 1, then rank 2, rank 3, and so on. The Wilcoxon rank-sum test statistic is the sum of the ranks for observations from one of the samples.

Table 2 shows the median (Q1–Q3) of the normalized test scores of the two groups in experiment 1. Only NormGain group shows significant difference (*p* < .01) in test scores between pretest and posttest. The test score of NormGain group is significantly larger (*p* < .001) than RevNormGain group.

## 5. Experiment  2: To Students without Prior Knowledge of LA

### 5.1. Participants

The dataset was collected over a period of four months during the fall and winter of 2014. The experiment and the manner of using data were approved by the Institution Review Board (IRB) of the National Chiao Tung University (Number NCTU – REC – 102 – 007). There were 25 students ranging in age from 23 to 26 (*M* = 24.0; SD = 0.8; 19 men and 6 women) participating in experiment 2. The participants were college students selected from a university in Taiwan, who just graduated from senior high schools, with normal or corrected-to-normal vision. The participants were required to have a basic understanding of high-school algebra and not have taken any college-level linear-algebra courses. They (1) were same in their age interval (SD = 0.8), (2) were same in domain knowledge they had, (3) were same in their college major, (4) were same in the length of time elapsing since they first learned the basic Algebra, (5) were enrolled in the same class of LA during the experiment, and (6) were same in the LA text book used for the study in the class of LA. All the participants self-reported that they were nonsmoker, healthy, and with no history of brain injury and cardiovascular problems. Each participant took from two to three weeks to complete the study over multiple sessions.

### 5.2. Hypotheses

Specifically, the hypotheses that experiment 2 aimed to prove are as follows.

- H2.1: There was no significant difference (*p* < .05) between NormGain and RevNormGain group in their test scores before the use of Tempranillo, for students without prior knowledge of LA.
- H2.2: There was significant difference (*p* < .05) in the test scores of NormGain group before and after the use of Tempranillo, for students without prior knowledge of LA.
- H2.3: There was no significant difference (*p* < .05) in the test scores of RevNormGain group before and after the use of Tempranillo, for students without prior knowledge of LA.
- H2.4: There was significant difference (*p* < .05) between NormGain and RevNormGain group in their test scores after the use of Tempranillo, for students without prior knowledge of LA.

### 5.3. Results

**Figure 13:**
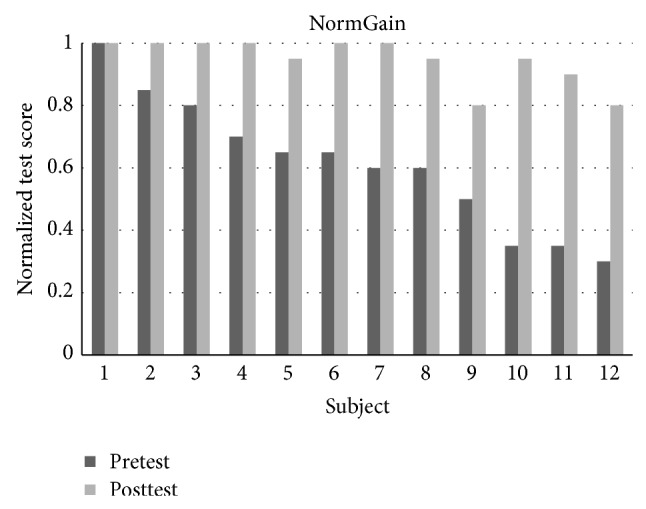
Normalized test score in experiment 2, NormGain group.

**Figure 14:**
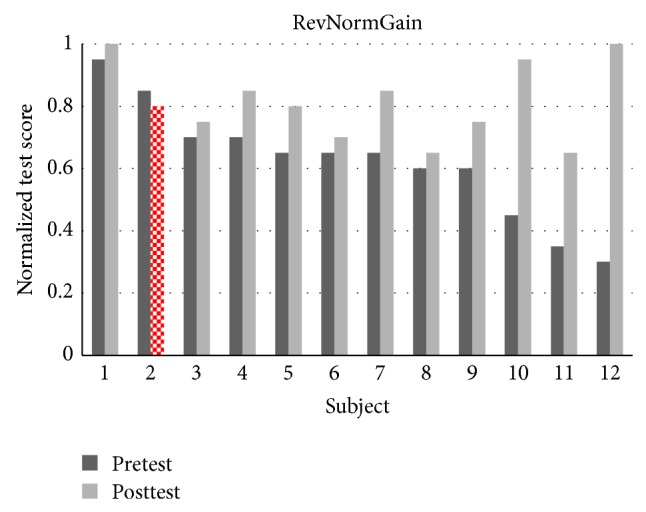
Normalized test score in experiment 2, RevNormGain group.

There were 25 students in experiment 2 and 24 students completed all topics. There were 12 students in the NormGain group and 12 students in the RevNormGain group. All of the test scores were normalized in the range of [0,1].  Figure 13 illustrates normalized test score of the students in NormGain group and Figure 14 illustrates normalized test score of the students in RevNormGain group.

**Figure 15:**
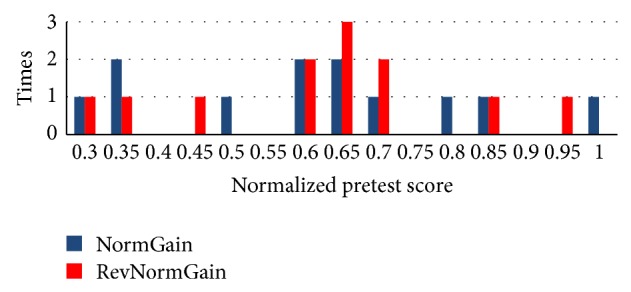
Normalized pretest score between two groups in experiment 2.

The histograms of students' pretest scores between two groups are shown in Figure 15.

**Figure 16:**
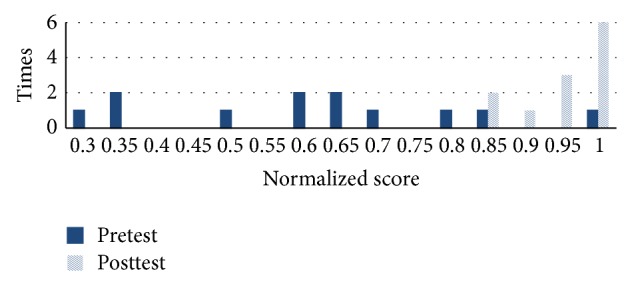
Normalized test score in experiment 2, NormGain group.

**Figure 17:**
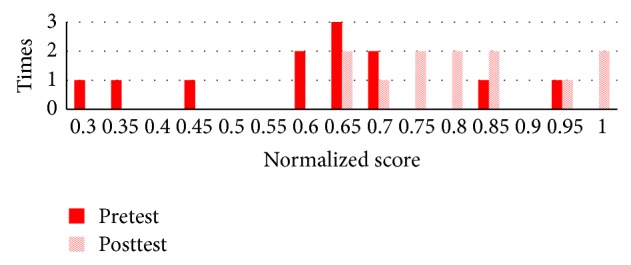
Normalized test score in experiment 2, RevNormGain group.

As shown in Figures 16 and 17, Figure 16 compares pretest and posttest scores of NormGain group.  Figure 17 compares pretest and posttest scores of RevNormGain group.

Table 3 shows the median (Q1–Q3) of the normalized test scores in experiment 2. There is no significant difference in pretest scores between the two groups. The test scores of both the two groups significantly (*p* < .01) increased after the learning session. The test score of NormGain group in posttest is significantly (*p* < .01) larger than the test score of RevNormGain group.

## 6. Discussions

**Figure 18:**
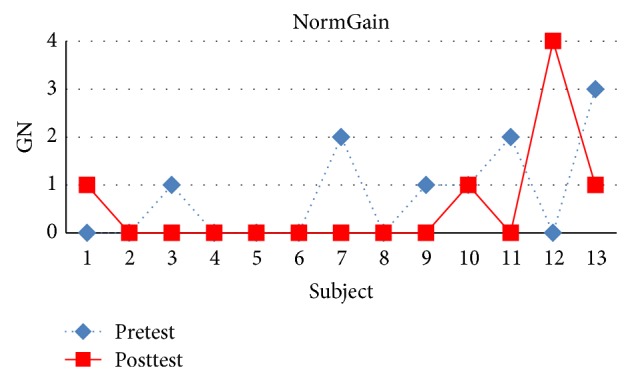
The GNs in experiment 1, NormGain group.

**Figure 19:**
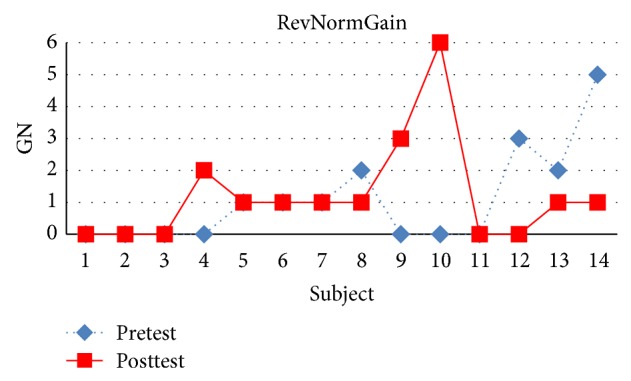
The GNs in experiment 1, RevNormGain group.

**Figure 20:**
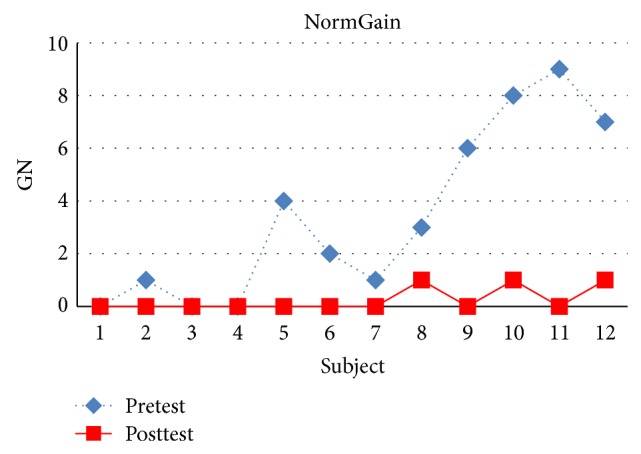
The GNs in experiment 2, NormGain group.

**Figure 21:**
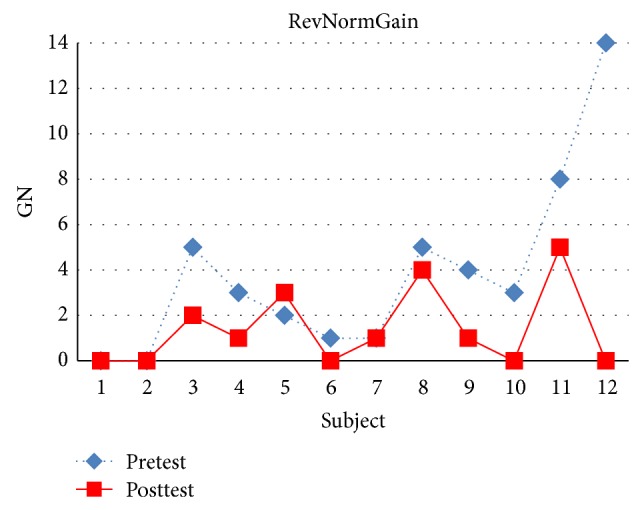
The GNs in experiment 2, RevNormGain group.

The results of experiment 1 support H1.1, H1.2, H1.3, and H1.4. And the results of experiment 2 support H2.1, H2.2, and H2.4 but not H2.3. To summarize, the results obtained indicate that Tempranillo is able to induce effective tutorial tactics in an online-learning manner, for both the students with or without the prior knowledge of LA. The induced tutorial tactics successfully increased the NLG of students in NormGain group (in both the experiments) and also suppressed the increase of NLG of students in RevNormGain group (only took place in experiment 1). A possible explanation of the failure of the RevNormGain tutorial tactics in suppressing the increase of NLG in experiment 2 (i.e., failed to prove H2.3) is that the presented materials (regardless of the present sequence) provide additional learning experiments that may influence the experimental results by renewing the information retrieving paths [33–35]. This result is in line with the finding in previous study [9], in which 64 college students participate in the experiment. On the other hand, H1.3 is supported because of that with the prior knowledge of LA (i.e., average pretest score = 0.875); those students left less room for improvement in regard to information retrieving paths. A student in the NormGain group in experiment 1 shows a decreased test score after the learning session (see Figure 8). Figure 9, furthermore, shows that in experiment 1 the number of students showing this pattern is larger in RevNormGain group compared to the NormGain group. However, in experiment 2 no student in the NormGain group shows similar pattern, whereas one student in the RevNormGain group showed this pattern. From our analysis of the number of guesses recorded during the pretest and posttest, we found that the pattern may be caused by students' guessing on the answers. The GNs that were collected from the questionnaire administered at the end of each problem in both the pretest and posttest are shown in Figures 18, 19, 20, and 21.

## 7. Conclusions

The aim of this study was to apply the GBML method for inducing tutorial tactics in an online-learning manner. Specifically, the XCS as a GBML algorithm which is able to optimize the interaction between the intelligent agent and the environment is used. The results indicate that Tempranillo is able to induce effective tutorial tactics in an online-learning manner. The induced tutorial tactics successfully increased the NLG of students (*p* < .01). The finding suggests that GBML should be a suitable approach for building an ITS with tutorial tactics. The future work may discuss other payoff function settings of the GBML methods to improve the learning speed of the agent. The exploration of the built models' generality to other learning domains and types of tutors should also be an important area of future work.

## Figures and Tables

**Table 1:** Relationship between state and module.

Module	Attribute
Student model	Pretest score
Expert knowledge	Topic ID
Expert knowledge	The number of given conceptual understanding (**D**_1_)
Expert knowledge	The number of given procedural knowledge (**D**_2_)
Expert knowledge	The number of given problem-solving (**D**_3_)
User interface	Subject ID
User interface	**D**_2_ answered correctly
User interface	**D**_3_ answered correctly
User interface	**D**_2_ + **D**_3_ answered correctly
User interface	The period from the system's login to logout time
User interface	SAM^∗^ valence (1–9)
User interface	SAM^∗^ arousal (1–9)
User interface	Engagement level (Csikszentmihalyi's flow theory)

^∗^Self-Assessment Manikin (SAM) is an affective rating system devised by Lang [23] to acquire the affective ratings.

**Table 2:** The median (Q1–Q3) of the normalized test scores of the two groups in experiment 1.

Test	Student group		*p* value
	NormGain	RevNormGain	
Pretest	0.9	0.875	0.55
	(0.775–0.95)	(0.7875–0.9125)	

Posttest	0.95	0.875	(*p* < .001)
	(0.95–1)	(0.7875–0.9125)	

*p* value	.008	1	

**Table 3:** The median (Q1–Q3) of the normalized test scores in experiment 2.

Test	Student group		*p* value
	NormGain	RevNormGain	
Pretest	0.625	0.65	.887
	(0.3875–0.775)	(0.4875–0.7)	

Posttest	0.975	0.8	.001
	(0.9125–1)	(0.7125–0.925)	

*p* value	.003	.004	
